# Synovial Fluid Immune Cell Composition Following Intraarticular Fracture May Contribute to Posttraumatic Osteoarthritis

**DOI:** 10.3390/ijms252212037

**Published:** 2024-11-09

**Authors:** Alexandra Hunter Aitchison, Nicholas B. Allen, Conor N. O’Neill, Lindsey G. Droz, Prekshaben Patel, Albert T. Anastasio, Rachel M. Reilly, Christian A. Pean, Malcolm R. DeBaun, James A. Nunley, Samuel B. Adams

**Affiliations:** 1 Department of Orthopaedic Surgery, Duke University Health System, Durham, NC 27710, USA; alexandra.aitchison@duke.edu (A.H.A.); nicholas.allen@duke.edu (N.B.A.); conor.n.oneill@duke.edu (C.N.O.); lindsey.g.johnson@duke.edu (L.G.D.); albert.anastasio@duke.edu (A.T.A.); rachel.reilly@duke.edu (R.M.R.); christian.pean@duke.edu (C.A.P.); malcolm.debaun@duke.edu (M.R.D.); james.nunley@duke.edu (J.A.N.); 2 Duke Immune Profiling Core, Duke University Health System, Durham, NC 27710, USA; preksha.patel@duke.edu

**Keywords:** intra-articular ankle fracture (IAF), post-traumatic osteoarthritis (PTOA), synovial fluid fracture hematoma (SFFH), immune cell profiling, flow cytometry, T cell subsets, inflammatory response, cartilage damage, cytokines, adaptive immune response

## Abstract

Intra-articular ankle fracture (IAF) often leads to post-traumatic osteoarthritis (PTOA), resulting in significant long-term morbidity. While previous research has focused on the inflammatory cytokines and matrix metalloproteinases within the synovial fluid fracture hematoma (SFFH), the immune cell populations within SFFH that contribute to PTOA development remain underexplored. This study aimed to characterize the immune cell populations in SFFH to better understand their role in the inflammatory response and potential for inducing lasting cartilage damage. Twenty-four patients with IAF underwent surgical ankle aspiration to collect SFFH, which was analyzed using polychromatic flow cytometry. The analysis revealed that 72.8% of the CD45+ cells were lymphocytes, predominantly CD3+ T cells (76.5%), with 42.1% being CD4+ and 39.2% CD8+ T cells. Additionally, monocytes accounted for 21.2% of CD45+ cells, with small populations of natural killer cells and myeloid-derived suppressor cells also present. These findings emphasize the predominance of T cells, particularly CD4+ subsets, in the immune response following IAF. Understanding these dynamics is essential for developing targeted interventions to prevent PTOA. Future research should focus on elucidating the specific roles of these immune cell populations in PTOA progression and exploring potential therapeutic strategies.

## 1. Introduction

Traumatic injuries to articular joints, such as intra-articular ankle fractures (IAF), frequently lead to post-traumatic osteoarthritis (PTOA), resulting in long-term disability and pain [1,2,3]. Classic arthritis thinking delineated inflammatory arthritis, such as rheumatoid arthritis, from degenerative arthritides, such as osteoarthritis and PTOA [1,4,5]. However, osteoarthritis and PTOA are increasingly being thought of as an inflammatory process as well [6,7]. Inflammation is now recognized as both a normal aspect of the biological response to articular injury and a pathological mechanism in arthritis development [2,7,8]. Synovial fluid isolated from patients after IAF has been shown to elicit downregulation of chondrogenic gene expression and decrease cell viability [9,10]. Previous research has found that anti-inflammatory therapeutic agents were able to modulate this response—albeit only partially [11]. In order to optimize therapeutic strategies for PTOA prevention, it is imperative to understand the entirety of the intraarticular environment following IAF.

The synovial fluid fracture hematoma (SFFH) that forms after IAF contains three main components including heme, inflammatory mediators, and immune cells; all of which may contribute to PTOA development. Short term exposure to blood causes lasting cartilage damage and increased heme breakdown products are known to greatly increase risk of developing arthritis [12,13,14,15,16]. Levels of pro-inflammatory cytokines and chemokines within the SFFH have been extensively characterized in the setting of IAF [5,17,18,19,20]. Biomarkers, including IL1-RA, IL-6, IL-8, IL-10, MMP-1, MMP-3, and MMP-9, are among most consistently elevated factors found in the SFFH following IAF [21]. The levels of these factors fluctuate during the acute phase of injury and remain present years after the fracture has healed suggesting a role of a lingering population of immune cells [22,23,24].

In rheumatoid arthritis, synovitis—characterized by synovial hypertrophy and infiltration of adaptive immune cells such as T cells and macrophages—drives the inflammation that ultimately leads to joint destruction [25,26,27]. Similarly, previous studies have identified mononuclear cells within the synovium of patients with idiopathic osteoarthritis (OA), including T cell infiltration in those with advanced OA undergoing joint replacement [28]. Lymphatic aggregates containing T cells have also been observed in the synovium during the early stages of OA, albeit to a lesser extent [29]. Despite the recognized role of the immune response in chronic inflammation and the pathogenesis of arthritides, the specific immune cell subsets contributing to early inflammation following IAF remain inadequately characterized. To date, only one small study involving six patients has investigated the immune population present after IAF [30]. The present study aims to expand the existing literature by providing a detailed immune cell profile of the SFFH following IAF in a large cohort of patients. This will help to better understand the inflammatory environment and possible mechanisms that contribute to the development of PTOA, ultimately informing future prevention strategies.

## 2. Results

Synovial fluid samples were successfully collected from twenty-four patients (15 females, 9 males) with an average age of 50.7 years (Table 1). The majority of the fractures from which SFFH was obtained were trimalleolar or bimalleolar, and the time from injury to SFFH collection averaged 12.2 days (range 4–28) (Table 1).

**Figure 1:**
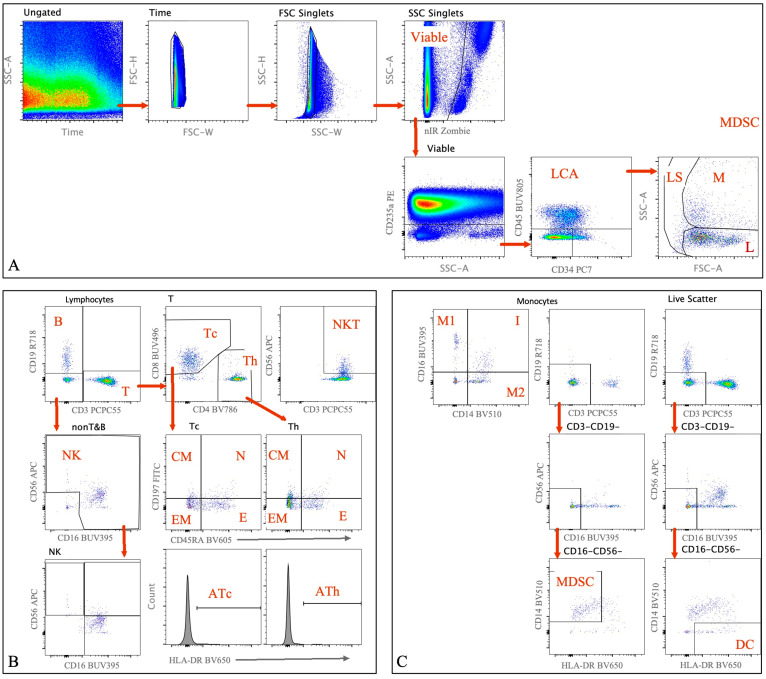
Polychromatic Flow Cytometry. Representative density plots and corresponding gating strategy used for cell population identification. Example obtained from one patient sample of SFFH. Sequential 2-dimensional hierarchical gating used to access subset of Monocytes, Lymphocytes, Myeloid-Derived Suppressor, and Dendritic cells. Sequential 2-dimensional gates are drawn using black text on the axes labels, bold black text above each plot indicates parent population (gate), red arrows indicate parent and child gates, bold red text is used to label populations identified by each respective plot. (**A**) Gates used in Pre-processing analysis include Time (Time versus FSC) to remove clogs/air bubbles, FSC and SSC singlets (FSC-W versus FSC-H and SSC-W versus SSC-H) to remove aggregates, Dead Cell exclusion using Zombie gating, CD235a used to remove red blood cells contamination, followed by identifying immune cells using CD45+ and Scatter gates to identify Lymphocytes (low FSC & SSC) and Monocytes (high FSC & SSC). (**B**) Lymphocytes were identified using scatter gating (FSC versus SSC), subsets of Lymphocytes were identified as follows: T cells (CD3+, CD3 versus CD19), B cells (CD3-CD19+), NK cells (CD3-CD19-CD16+56+), and NKT cells (CD3+CD56+). Cytotoxic T cells (Tc) and Helper T cells (Th) subsets of T Lymphocytes were identified as CD4-CD8+ (Tc) and CD4+CD8- (Th) using CD4 versus CD8 gated on T cells. Subsets of Th and Tc were identified as Naïve (N) (CD45RA+CCR7+); Central Memory (CM)(CD45RA-CCR7+); Effector Memory (EM) (CD45RA-CCR7-); and TEMRA (E) (CD45RA+CCR7-). Activation of Th (ATh) and Tc (ATc) was determined by HLA-DR+. (**C**) Monocytes were identified using scatter gating (FSC versus SSC), CD14 versus CD16 was used to identify subsets of Monocytes: CD14+CD16- (Classical Monocytes, M1), CD14-CD16+ (Non-Classical Monocytes, M2), CD14+CD16+ (Intermediate Monocytes). Myeloid-Derived Suppressor cells (MDSCs) were identified as Lin-(CD3-CD19-CD16-CD56-)HLA-DRlowCD14+ [31]. Dendritic Cells (DC) were identified as Lin-(CD3-CD19-CD16-CD56-) HLA-DR+.

Flow cytometry was performed using a panel of markers as outlined previously. The representative gating strategy used to analyze immune cell populations within the SFFH can be found in Figure 1. Note that this figure represents plots from one of the twenty-four samples included in this study.

The majority of the cells were adaptive immune cells, dominated by CD3+ T cells (76.5%) and a smaller population of CD19+ B cells (2.3%). Natural killer cells, monocyte lineage cells, myeloid-derived suppresser cells (MDSC), and dendritic cells (DC) were also detected within the SFFH in smaller fractions (Table 2).

**Figure 2:**
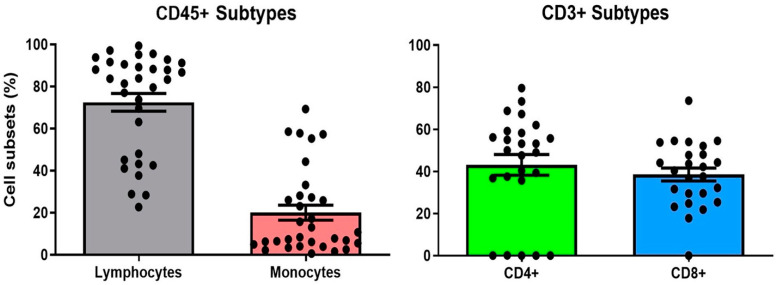
Immune Profile of SFFH. Bar charts with dots representing percent presence of CD45+ gated subgroups (left) and CD3+ gated (right) subgroups within individual samples of SFFH. Error bars represent standard error of the mean.

Within the CD3+ T cell population, there was a substantial presence of both CD4+ and CD8+ T cells. CD4+ T cells accounted for an average of 42.1% of the CD3+ population, while CD8+ T cells made up an average of 39.2% (Figure 2). This distribution suggests a relatively balanced adaptive immune response within the synovial fluid following an ankle fracture. These CD3+ T cells were further divided into multiple subsets, which were well-represented across different stages of differentiation as can be seen in Table 2. Other CD45+ cell subsets, including B cells, NK cells, monocyte, MDSCs, and DCs are present indicating a diverse immune profile. Among monocytes, the classical subtype was predominant, contributing to the inflammatory milieu within the synovial fluid.

**Figure 3:**
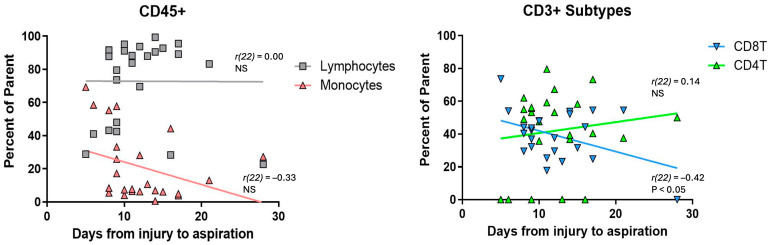
Time dependent immune profile of SFFH. Scatter plots showing trends in percent of CD45+ (left) and CD3+ (right) subtypes depending on time from injury to SFFH aspiration. NS; not significant.

To further investigate temporal difference in the immune response, the relative abundance of CD45+ and CD3+ subsets was plotted against time from injury to SFFH collection (Figure 3).

The lymphocyte population remains stable over time (*r(22)* = 0.00, n.s.), while there is a non-significant trend towards a decrease in monocytes as time from injury increases (*r(22)* = −0.33, n.s.). Within the CD3+ lymphocytes, the CD4+ T cell population shows a non-significant trend towards increasing with time from injury (*r(22)* = 0.14, n.s.), whereas the population of CD8+ T cells significantly decreases as time from injury increases (*r(22)* = −0.42, *p* = 0.04). n.s.; not significant.

## 3. Discussion

This study is the largest to date that successfully profiles the immune cell population within human-derived synovial fluid after an ankle fracture. Flow cytometry analysis revealed the presence of a diverse immune cell landscape within the synovial fluid following intra-articular fractures. In addition to the predominance of CD4+ and CD8+ T cells, several differentiated subsets of both helper and cytotoxic T cells were identified, reflecting a range of activation and memory stages. The high presence of effector and central memory cells suggests a prolonged immune response post-injury. Non-T cell populations, including monocytes, NK cells, myeloid-derived suppressor cells (MDSCs), and DCs were also present, albeit in smaller proportions, further adding to the complexity of the immune response, suggesting multiple pathways that could contribute to post-traumatic osteoarthritis (PTOA) development. Understanding these dynamics is crucial for developing targeted therapies to prevent PTOA.

Building upon the foundational work by Furman et al., who first characterized immune populations in the SFFH of six patients following IAF, our study involving 24 patients provides a more extensive view of immune cell dynamics. We observed a slightly higher percentage of CD3+ T cells (77% compared to 63%) and a smaller population of CD19+ B cells (2% compared to 10%). The CD4+ and CD8+ T cell subsets among CD45+ cells showed similar prevalence (42% and 39%, respectively) compared to Furman’s findings (49% and 36%, respectively), reinforcing the notion that these immune cells play a central role in the post-injury inflammatory response. This study also expanded on previous work by incorporating additional immune populations, providing a more comprehensive characterization of the immune landscape following acute IAF.

The high proportion of CD3+ T cells found in both these studies suggest these cells are central to the inflammatory response following IAF. T cells, particularly the CD4+ and CD8+ subsets, play a key role in regulating the immune response in arthritis, where CD4+ T cells often promote inflammation through cytokine production and activation of other immune cells, while CD8+ T cells can contribute to tissue damage and sustained inflammation [32,33]. The balance between these T cell subsets could influence the progression to PTOA, as their differing roles in inflammation and tissue repair are crucial to disease outcomes. This is consistent with findings in idiopathic osteoarthritis (OA), where T cell infiltration and the formation of lymphatic aggregates within the synovium have been observed, particularly in advanced stages of the disease. These immune cell activities in OA highlight a similar pathogenic mechanism, where chronic inflammation driven by T cells contributes to joint degeneration. The parallels between immune responses in idiopathic OA and post-traumatic osteoarthritis (PTOA) further emphasize the central role of T cells in mediating inflammatory processes that lead to cartilage damage. Although less predominant, monocytes may contribute to the inflammatory environment by differentiating into macrophages, which infiltrate the synovium and are known to play roles in both initiating and resolving inflammation [5]. This T cell dominance and macrophage presence is further influenced by the cytokine environment within the joint, which is known to recruit and activate various immune cells.

The inflammatory response following an intra-articular ankle fracture is characterized not only by the presence of specific immune cell populations but also by the profile of cytokines and chemokines in the synovial fluid. Previous studies have demonstrated that pro-inflammatory cytokines, such as IL-1β, TNF-α, and IL-6, are significantly elevated in the synovial fluid following acute articular fractures which is crucial in recruiting immune cells to the injury site and sustaining the inflammatory response. Both IL-1β and TNF-α recruit and activate T cells and macrophages at the injury site, and they also stimulate IL-6 production in osteoblasts [34], which correlates with our finding of high percentages of CD3+ cells within the SFFH. TNF-α and IL-6 are also implicated as pathogenic factors in immune-related bone disorders, including rheumatoid arthritis and postmenopausal osteoporosis, further highlighting their pathogenic potential to bone [35].

The interplay between these cytokines and chemokines and the immune cell populations they recruit underscores the complexity of the inflammatory response following IAF. For example, IL-1β has been suggested as a key driver in the early immune response, promoting the migration and activation of these cells. Pharmaceutical interventions targeting IL-1β, such as IL-1Ra (anakinra), have been studied in the context of PTOA with mixed results. Allen et al. demonstrated that targeting IL-1β with inhibitors like IL-1Ra (anakinra) partially reduced cartilage damage in explants cultured with SFFH. Another study found that treatment with IL-1Ra decreased the initial inflammatory burden after intraarticular fracture in an animal model but did not improve prevent arthritis development measured by ORASI scores 56 days post injury. A contradicting study showed treatment with IL-1Ra successfully prevented PTOA development in mice [11,30,36,37]. While targeting IL-Iβ and thereby reducing infiltration of immune cells shows some benefit in initial immune response and cartilage damage, the variable effect on development of PTOA highlights the multifactorial nature of the initial immune response after intraarticular fracture.

One aspect of our study is the observation of changes in immune cell populations over time within the SFFH following IAFs. While Furman et al. looked at immune cells at a single time point and some studies have looked at immune cell profiles in early versus late OA, there has not been focus on how these populations change after an intraarticular injury that may trigger the development of arthritis. Our study offers a view of how these immune cells, particularly CD4+ and CD8+ T cells, fluctuate over the first few weeks post-injury. Although our timeframe is relatively short, these findings provide a useful glimpse into the early immune response that could play a role in the development of PTOA. By exploring these time-dependent changes, our study adds to the understanding of the immune environment following IAF, even if further research is needed to fully map these dynamics over a longer period.

Furthermore, it is important to understand how immune cell populations within the joint influence the post fracture environment over time and how they contribute to known fluctuations in cytokines. Beyond the acute injury phase, the sustained presence of pro-inflammatory cytokines is a key driver of chronic inflammation, which plays a crucial role in the development of PTOA. Research has shown that cytokines such as IL-6, along with other immune mediators, remain elevated in synovial fluid well past the acute phase and even after fracture healing is complete. IL-6, which is notably produced by a variety of cells including macrophages and the TH2 subset of CD4+ T cells, acts as a pro-inflammatory mediator downstream of the IL-1β pathway in chondrocytes and synovial tissues. It contributes to reduced matrix production and increased matrix degradation. The persistent elevation of IL-6 suggests that immune cell populations are instrumental in maintaining this ongoing inflammatory environment. Our findings align with this, showing temporal shifts in immune cell subsets over the first 28 days post-injury. Specifically, within the CD3+ T cell population, there is a significant decrease in the percentage of CD8+ T cells over time, with an increasing trend in the percentage of CD4+ T cells, indicating a dynamic shift in the immune landscape as the injury heals.

While this study represents the largest to date, several limitations must be acknowledged. Firstly, there was variability in patient details, including age, fracture pattern, and the timing of synovial fluid aspiration. These factors could influence the immune profile, yet, outside of time, we did not analyze how these variables impacted our findings. Additionally, we did not account for comorbidities or medications that could potentially alter immune responses or cytokine levels, which might have introduced confounding factors. Secondly, due to limited sample volumes, we prioritized immune cell population characterization, and as a result, simultaneous cytokine analysis was not performed. However, as cytokines play a critical role in mediating the immune response, this omission limits our ability to directly correlate immune cell presence with cytokine activity. Future research should aim to correlate immune cell profiles with cytokine levels from the same patients to further elucidate the inflammatory pathways driving PTOA. Thirdly, the lack of long-term follow-up precludes us from determining whether the immune profiles observed are predictive of PTOA development. Identifying which patients developed PTOA and comparing their immune profiles to those who did not could provide significant insights into the pathogenic immune responses driving the disease. Lastly, our study is inherently limited by the sample size, despite being the largest of its kind, and the results may not capture the complex immune environment in the joint following IAF not be fully generalizable to all patients.

Future research should investigate the functional roles of specific immune cell subsets in the setting of fracture and their interplay with inflammatory cytokines to provide deeper insights into the mechanisms driving PTOA development and pave the way for targeted therapies. Additionally, longitudinal studies to monitor changes in immune cell populations alongside cytokine levels in human synovial fluid over time following IAF are imperative to understand the temporal dynamics of the inflammatory response and identify critical windows for therapeutic intervention. Although we did not analyze immune cell populations in peripheral blood in the current study, future studies should also investigate this comparison to better understand systemic versus localized immune responses in post-traumatic osteoarthritis. Finally, long term patient follow-up to determine the immune profile of those patients that developed PTOA compared to those that did not may facilitate identification of a pathogenic immune signature versus one that leads to healing.

## 4. Materials and Methods

### 4.1. Sample Collection

After IRB-approval, synovial fluid was obtained from twenty-four eligible patients with acute IAF. Patient demographics, including age, sex, and fracture type, were recorded. Eligibility criteria included patients over 18 years old with no prior ankle surgery who sustained primary, closed ankle fractures and underwent operative reduction and internal fixation (ORIF). Exclusion criteria included non-traumatic ankle fractures (e.g., pathologic, neuropathic), open fractures, ORIF for nonunion, and fractures treated nonoperatively. Synovial fluid samples were collected at the time of surgical fixation using a sterile aspiration technique. Using an anteromedial approach to the ankle joint, a 16-gauge needle was inserted, and the contents of the joint were aspirated and immediately transferred to Eppendorf tubes and kept on ice.

### 4.2. Polychromatic Flow Cytometry

Freshly collected synovial fluid from acute ankle injuries were collected in a 50 mL conical and stored on ice during transport to the Duke Immune Profiling Core (DIPC) for processing, staining, and acquisition. During sample processing, red blood cell pellets were observed in all samples. A 1 × Lyse Solution (BD Biosciences, Franklin Lakes, NJ, USA) was used to lyse RBC contamination following manufacture recommendations. After lysing, up to 45 mL cold Dulbecco’s PBS (DPBS) was added to each sample and samples were centrifuged at 350× *g* for 10 min at 4 °C. The supernatant was decanted, and the samples were resuspended in the residual volume. All cells were added to a 12 × 75 polypropylene tube for staining along with 3 mL cold DPBS. The samples were centrifuged at 350× *g* for 5 min at 4 °C. The supernatant was again decanted, and the samples were resuspended in the residual volume. Near IR Zombie dye (nIR, BioLegend, San Diego, CA, USA) was reconstituted using provided DMSO and aliquoted into 20 µL aliquots which were stored at −20 °C. A 1:10 working stock of the nIR Zombie dye was prepared using cold DPBS and then added to the cells along with Human TruStain FcX (BioLegend) for a final staining volume of 100 µL. Samples were incubated at room temperature (18–22 °C) for 15 min. Samples were washed in 2 mL cold Wash Buffer (DPBS + 0.5% FBS) and then surface stained on ice for 30 min in a final volume of 150 µL using the antibodies and fluorophores listed in Table 3. Samples were then washed in 3 mL cold Wash Buffer and the samples were centrifuged at 350× *g* for 5 min at 4 °C. The supernatant was decanted and resuspended in 150 µL cold 1% Formalin. Samples were acquired immediately after staining using a BD SORP Fortessa analyzer (BD Biosciences, Franklin Lakes, NJ, USA). BD anti-Mouse CompBeads were be used for fluorescence compensation. Gating and analysis regions were be established using batch control peripheral blood mononuclear cell samples (example in Figure 1). Each experimental sample was successfully processed for immune phenotyping, and viable CD45+ counts were reported for all samples. The resulting data were analyzed using FloJo software (version 10).

### 4.3. Statistical Analysis

Data representation and statistical analysis were performed using GraphPad Prism (version 10.3.01) software. Descriptive statistics including mean and standard deviations were used to describe the immune cell populations. Pearson’s correlation was used to assess the relationships between the time from injury and the proportions of monocytes, lymphocytes, and T cell subsets (CD4+ and CD8+). Correlation coefficients (r), along with *p*-values, were reported to determine the significance of these relationships, with a significance threshold set at *p* < 0.05. Data are presented as the mean ± standard deviation.

## 5. Conclusions

This study provides a detailed characterization of the immune cell populations in the SFFH following acute IAF. The predominant presence of T cells, particularly the CD4+ and CD8+ subsets, suggests their significant role in the inflammatory response and subsequent PTOA development. The correlation between elevated pro-inflammatory cytokines and the presence of specific immune cell populations highlights the complex interplay driving post-injury inflammation. These findings indicate that early modulation of T cell-mediated immune responses, particularly those involving CD4+ and CD8+ subsets, could offer a therapeutic approach to mitigate the inflammatory environment following intra-articular fractures. Future research should explore the potential of immunomodulatory treatments to accelerate healing and prevent the progression of post-traumatic osteoarthritis, offering new strategies to protect joint health in patients with IAF.

## Figures and Tables

**Table 1:** Patient Demographics and Injury Details.

Variable	N (%)/Mean ± SD
Age (years)	50.7 ± 15.8
Sex	
Female	15 (63%)
Male	9 (37%)
Fracture Type	
Trimalleolar	10 (42%)
Bimalleolar	10 (42%)
Fibular (+Deltoid Ligament Tear)	2 (8%)
Pilon	2 (8%)
Time from injury to aspiration (days)	12.2 ± 4.9

**Table 2:** Percentage Distribution of Immune Cell Subsets Analyzed by Flow Cytometry. TEMRA; Terminal effector memory T cells, NKT; Natural Killer-like T cells, NK; Natural Killer cells, MDSC; Myeloid-Derived Suppressor Cells, DC; Dendritic Cells.

Parent	Subset/Marker	Mean Percentage (%) ± SD
CD45+	Lymphocytes (SSC low FSC low)	72.8 ± 25.2%
Lymphocytes	T Cells (CD3+)	76.5 ± 14.5%
CD3+ T Cells	Helper T Cells (CD4+)	42.1 ± 24.5%
CD4+ Th Cells	- Naïve (CD45RA+CCR7+)	10.8 ± 11.9%
CD4+ Th Cells	- Central Memory (CD45RA-CCR7+)	27.0 ± 26.6%
CD4+ Th Cells	- Effector Memory (CD45RA-CCR7-)	42.4 ± 32.5%
CD4+ Th Cells	- TEMRA (CD45RA+CCR7-)	7.9 ± 15.5%
CD4+ Th Cells	- Activated Helper T Cells (HLA-DR+)	4.9 ± 5.5%
CD3+ T Cells	Cytotoxic T Cells (CD8+)	39.2 ± 15.4%
CD8+ Tc Cells	- Naïve (CD45RA+CCR7+)	13.3 ± 11.7%
CD8+ Tc Cells	- Central Memory (CD45RA-CCR7+)	11.8 ± 15.1%
CD8+ Tc Cells	- Effector Memory (CD45RA-CCR7-)	36.0 ± 23.4%
CD8+ Tc Cells	- TEMRA (CD45RA+CCR7-)	34.8 ± 23.2
CD8+ Tc Cells	- Activated Cytotoxic T Cells (HLA-DR+)	22.7 ± 14.9%
CD3+ T Cells	NKT Cells (CD56+)	8.4 ± 6.5%
Lymphocytes	B Cells (CD19+)	2.3 ± 2.9%
CD3-CD19-	NK Cells (CD16+CD56+)	59.4 ± 27.4%
CD45+	Monocytes (SSC high FSC low)	21.2 ± 21.0%
Monocytes	Classical (CD14++CD16-)	36.3 ± 18.5%
Monocytes	Intermediate (CD14++CD16+)	19.2 ± 16.0%
Monocytes	Non-Classical (CD14+CD16++)	17.3 ± 20.9%
Monocytes	MDSCs (Lin-CD14+DR^low^)	15.1 ± 21.9%
Live Scatter (CD45+CD235a-CD34-)	DCs (Lin-DR+)	2.6 ± 3.3%

**Table 3:** Monoclonal Antibodies and Corresponding Fluorophores Used for the Flow Cytometric Surface Staining of Synovial Fluid Samples.

mAb	Fluorophore	Clone
nIR Zombie		n/a
CD197 (CCR7)	FITC	G043H7
CD3	PerCP-Cy5.5	SK7
CD14	BV510	M5E2
CD45RA	BV605	HI100
HLA-DR	BV650	L243
CD4	BV786	Sk3
CD56	APC	MHCD56
CD19	R718	HIB19
CD235a	PE	HI264
CD34	PE-Cy7	8G12
CD16	BUV395	3G8
CD8	BUV496	SK1
CD45	BUV805	HI30

## Data Availability

The data presented in this study are available on request from the corresponding author.
